# Supplementary material for: Inverted shear-strain magnetoelastic coupling at the Fe/BaTiO3 interface from polarised x-ray imaging
Source: Nat Commun. 2025 Sep 26;16:8445. doi: 10.1038/s41467-025-62978-2 (PMC12475209; doi:10.1038/s41467-025-62978-2)
Supplement: Supplementary file 1 — Supplementary Information [file 41467_2025_62978_MOESM1_ESM.pdf]

Supplementary Material
for
Inverted shear-strain magnetoelastic coupling at the Fe/$BaTiO_3$ interface from polarised x-ray imaging.

Francesco Maccherozzi,[1,*] Massimo Ghidini,[2,1,3] Mary Vickers,[3]
Xavier Moya,[3] Stuart A. Cavill,[4] Hebatalla Elnaggar,[5,6]
Anne D. Lamirand,[7] Neil D. Mathur,[3] and Sarnjeet S. Dhesi[1,†]

[1]*Diamond Light Source, Harwell Science and Innovation Campus, Didcot, OX11 0DE, UK*
[2]*Department of Mathematics, Physics and Computer Science, University of Parma, 43124 Parma, Italy*
[3]*Department of Materials Science, University of Cambridge, Cambridge, CB3 0FS, UK*
[4]*School of Physics, Engineering and Technology, University of York, York YO10 5DD, UK*
[5]*Debye Institute for Nanomaterials Science, Utrecht University, 3584 CA Utrecht, The Netherlands*
[6]*Institute of Mineralogy, Physics of Materials and Cosmochemistry, CNRS, Sorbonne University, 4 Place Jussieu, 75005 Paris, France*
[7]*Ecole Centrale de Lyon, INSA Lyon, CNRS, Universite Claude Bernard Lyon 1, CPE Lyon, INL, UMR5270, 69130 Ecully, France*

(Dated: July 18, 2025)

* francesco.maccherozzi@diamond.ac.uk
† sarnjeet.dhesi@diamond.ac.uk

I. EPITAXIAL GROWTH

Figure SM 1(a) shows a typical Low Energy Electron Diffraction (LEED) pattern from a clean $BaTiO_3$ (BTO) surface confirming the surface crystallinity. Figure SM 1(b) shows a typical LEED pattern after Fe deposition confirming epitaxial growth with $[110]_{Fe} \parallel [100]_{pc}$.

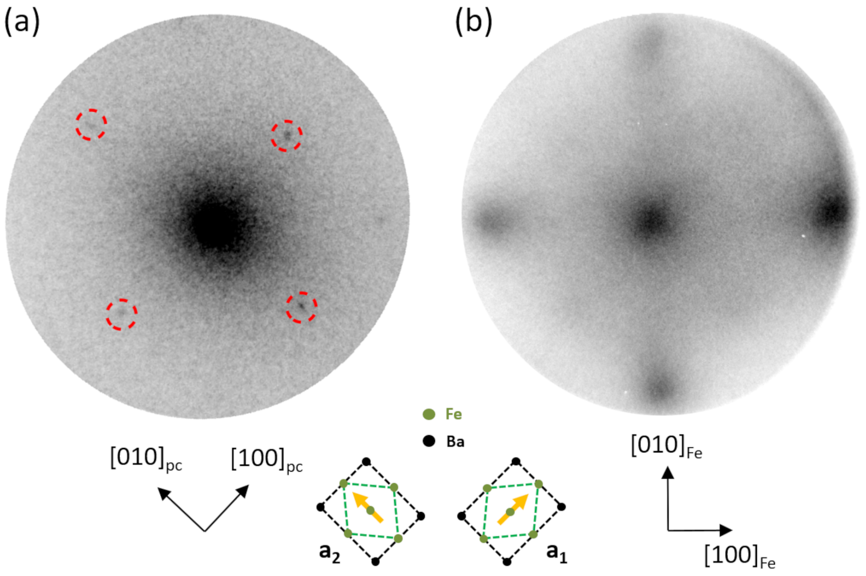

Figure SM 1. (a) LEED pattern from the cleaned BTO substrate, with broken red-line circles around the diffraction spots, and (b) from the Fe film. The electron kinetic energy, E_{kin}, was 21.4eV. The schematic shows crystallographic axes and the epitaxial relation of the Fe and BTO unit cells for two BTO Ferroelastic (FEL) domains.

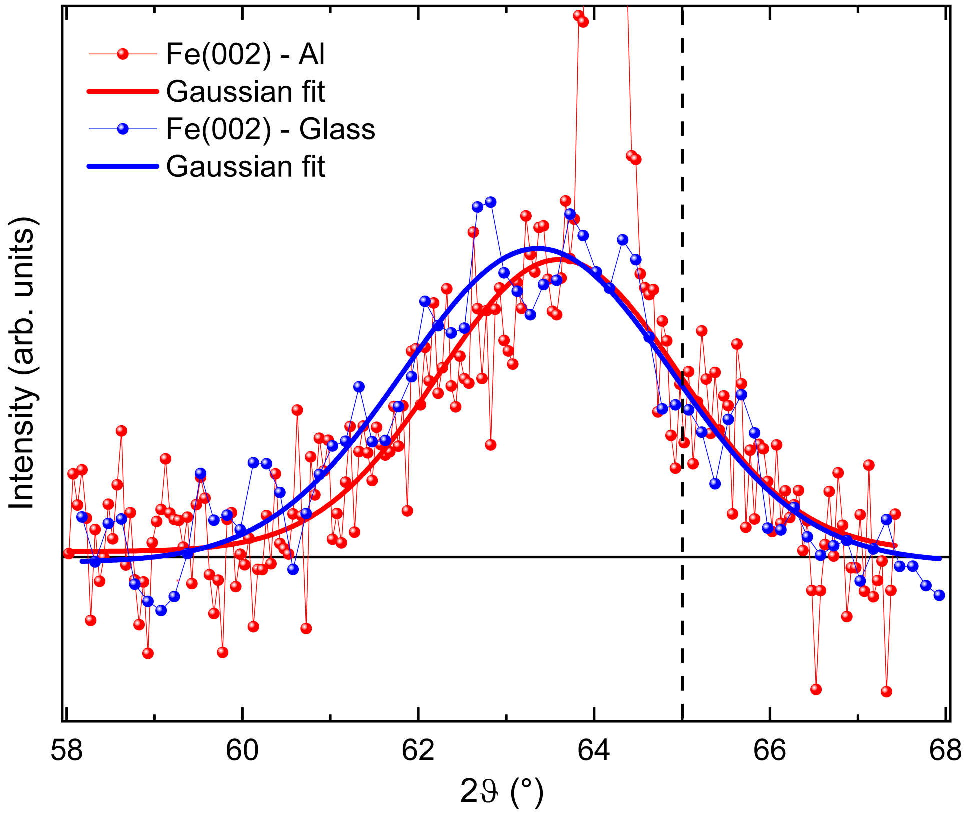

Figure SM 2. XRD 2θ scans showing a weak and broad (002) peak from the Fe film. The red data points are the summation of multiple scans taken at different azimuthal angles, with respect to the X-ray beam direction. The intense reflection at $\sim 64.2°$ arises from the Al sample holder and is absent in the single scan taken using a glass sample holder, shown as the blue data points. The solid red and blue lines show Gaussian fits to the data. The broken line indicates the expected position of the (002) peak for a fully relaxed Fe film.

The XRD pattern of the Fe film shows a single broad reflection (see Figure SM2) at a 2θ angle consistent with strained Fe (002). The out-of-plane interplanar spacing is under tension, and therefore consistent with the expected compression of the in-plane lattice parameter for epitaxial growth of Fe on BTO. XRD and LEED data are consistent as both give broad Fe peaks confirming that the Fe film grew epitaxially and is very thin. Using the Debye-Scherrer formula, the film thickness is estimated to be 2.8 ± 0.3 nm from the XRD data. Combining the thickness derived from XRD, with those derived from X-ray Reflectivity and from the *in situ* calibration, using a quartz crystal microbalance thickness monitor, gives an Fe film thickness of 2.2 ± 0.4 nm.

II. MIRROR ELECTRON MICROSCOPY

Figure SM 3(a) shows a Mirror Electron Microscopy (MEM) image of a clean BTO surface exhibiting FEL and ferroelectric (FE) domains. In MEM, probing electrons reach the sample surface with nearly zero kinetic energy and are sensitive to the local surface morphology and electrostatic potential [1], permitting the electronic surface charge topography to be mapped. The contrast arises from stray electric fields generated by the FE domains.

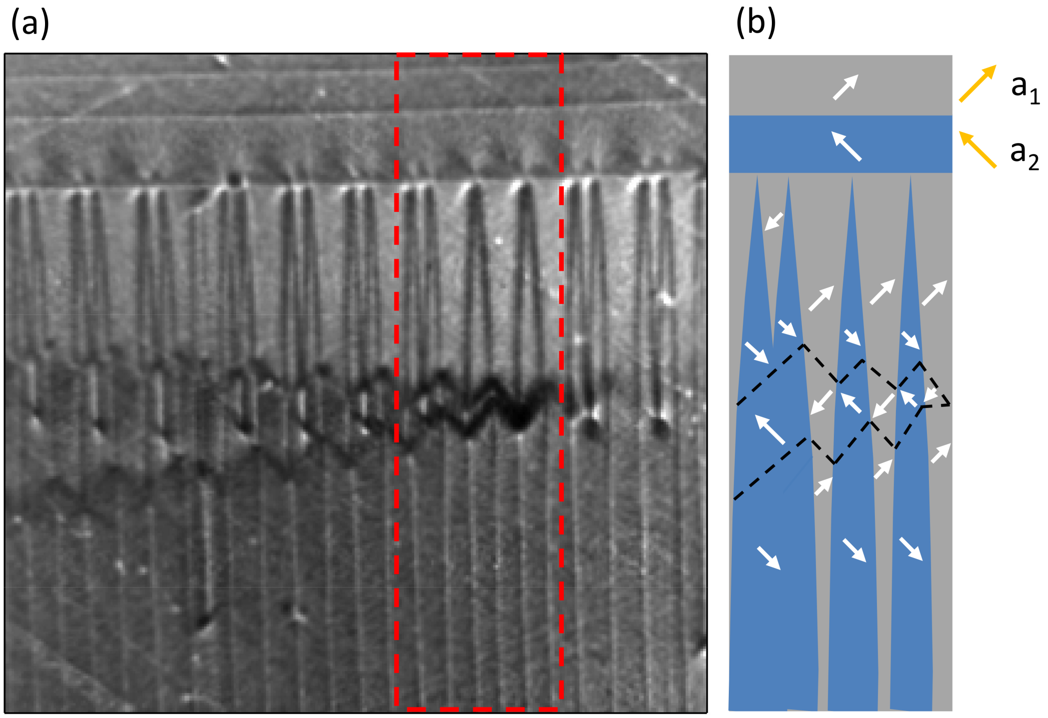

Figure SM 3. (a) MEM image of a clean BTO surface prior to Fe deposition (E_{kin} = 24.4 eV). (b) Schematic representation of the structure and orientation of the *in-plane* FEL and FE domains within the dashed red rectangle in (a). The a_1 and a_2 FEL domains are shown as blue and grey regions, with yellow arrows indicating the corresponding BTO c-axis direction. The local orientation of the FE polarisation is shown as the white arrows. It is not possible to determine the local FE polarisation direction with MEM, so that the case with all of the white arrows reversed would also be valid. The dashed black lines indicate the position of the 180° FE domain walls.

FEL domains boundaries are visible as lines [2] running along the $[1\bar{1}0]_{pc}$ direction in the upper section, and predominantly along the $[110]_{pc}$ direction in the lower section. The zig-zag dark lines are dynamically generated by the electron beam and move on the surface

in response to changes in E_{kin} (see SM Video 1). These zig-zag lines have been previously associated with the creation of mobile 180° FE domain walls [3]. Figure SM 3(b) shows a schematic representation of the MEM image with the FEL domains drawn as grey and blue areas and the local FE polarisation shown as red arrows. The 180° FE domain walls are shown as dashed black lines. Since the MEM image contrast arises from the surface electric polarisation, it indicates that the stoichiometry of the surface is preserved after the cleaning treatment. BTO surfaces prepared at higher temperatures exhibited no MEM contrast due to polarisation compensating surface contaminants.

III. MULTIPLET CALCULATIONS

Multiplet calculations were performed using QUANTY [4]. The Hamiltonian used was

$$\mathcal{H} = \mathcal{H}_{e-e} + \mathcal{H}_{SO} + \mathcal{H}_{CF}, \tag{1}$$

where $\mathcal{H}_{SO}$ is the spin-orbit coupling, $\mathcal{H}_{CF}$ is the crystal field potential and the electron-electron Hamiltonian, $\mathcal{H}_{e-e}$, is

$$\mathcal{H}_{e-e} = \sum_k f_k F^k + \sum_k g_k G^k, \tag{2}$$

$F^k(f_k)$ and $G^k(g_k)$ are the Slater-Condon parameters for the radial and angular operators of the direct and exchange Coulomb interactions, respectively. The radial integrals are obtained from atomic Hartree-Fock calculation scaled to 70% and 80% for valence and valence-core interactions, respectively, to take into account interatomic screening and charge mixing effects. The 80% reduction corrects the Hartree-Fock calculations to agree with atomic data, as shown by Cowan. The additional reduction to 70% is to take into account the effects of charge transfer. $\mathcal{H}_{SO}$ is of the form

$$\mathcal{H}_{SO} = \xi \sum_i l_i \cdot s_i, \tag{3}$$

where l_i and s_i are the one electron orbital and spin operators, respectively, and the sum over i is over all electrons.

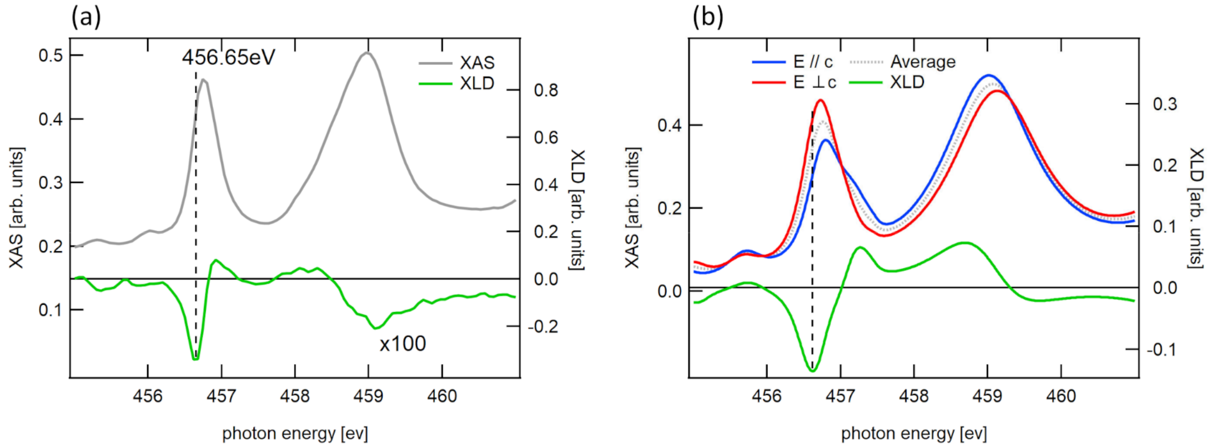

Figure SM 4. (a) BTO Ti-L_3-edge XAS and XLD spectra, (b) XAS and XLD multiplet simulations over the Ti L_3-edge

The prefactor ξ is an atom dependent constant derived from tabulated data. $\mathcal{H}_{CF}$ can be expressed as the sum of normalised spherical harmonics as

$$\mathcal{H}_{CF} = \sum_{k=0}^{4} \sum_{m=-k}^{k} A_{k,m} C_{k,m}(\theta, \phi), \tag{4}$$

Figure SM 4(a) shows a Ti L_3-edge X-Ray Absorption Spectroscopy (XAS) spectrum along with the X-Ray Linear Dichroism (XLD). Figure SM 4 (b) shows calculated Ti L_3-edge XAS spectra for **E** parallel and perpendicular to the c-axis along with the corresponding XLD spectrum. The multiplet calculations were done with $10D_q = 1.95eV$ and $D_t = 1.5meV$. Figure SM 5 shows experimental Fe $L_{2,3}$-edge spectra for left and right circularly polarised light along with the corresponding X-Ray Magnetic Circular Dichroism (XMCD) spectrum.

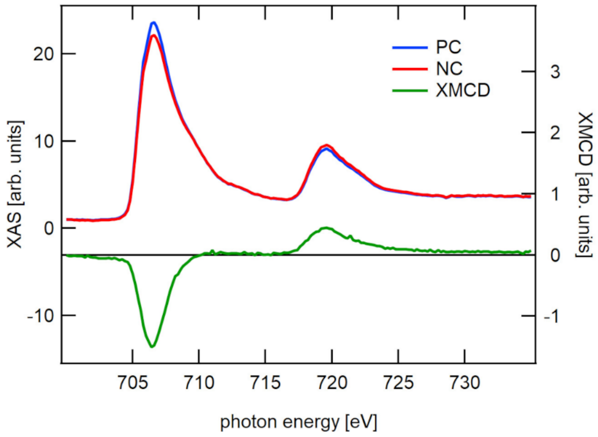

Figure SM 5. Fe $L_{2,3}$-edge XAS and XMCD spectra.

IV. FERROELASTIC DOMAIN IMAGING

Figure SM 6 shows XLD-PEEM images of the BTO a_1 and a_2 FEL domains for two orthogonal incoming light directions. A bright contrast indicates that the electric field vector (**E**) is perpendicular to the local BTO c-axis whilst a dark contrast indicates that **E** is parallel to the local c-axis. The XLD contrasts reverse with the 90° rotation of the incident light direction. For XLD-PEEM images recorded with **E** at 74° to the surface plane, no XLD contrast was detectable, indicating that the local c-axis of the a_1 and a_2 FEL domains lies in the surface plane.

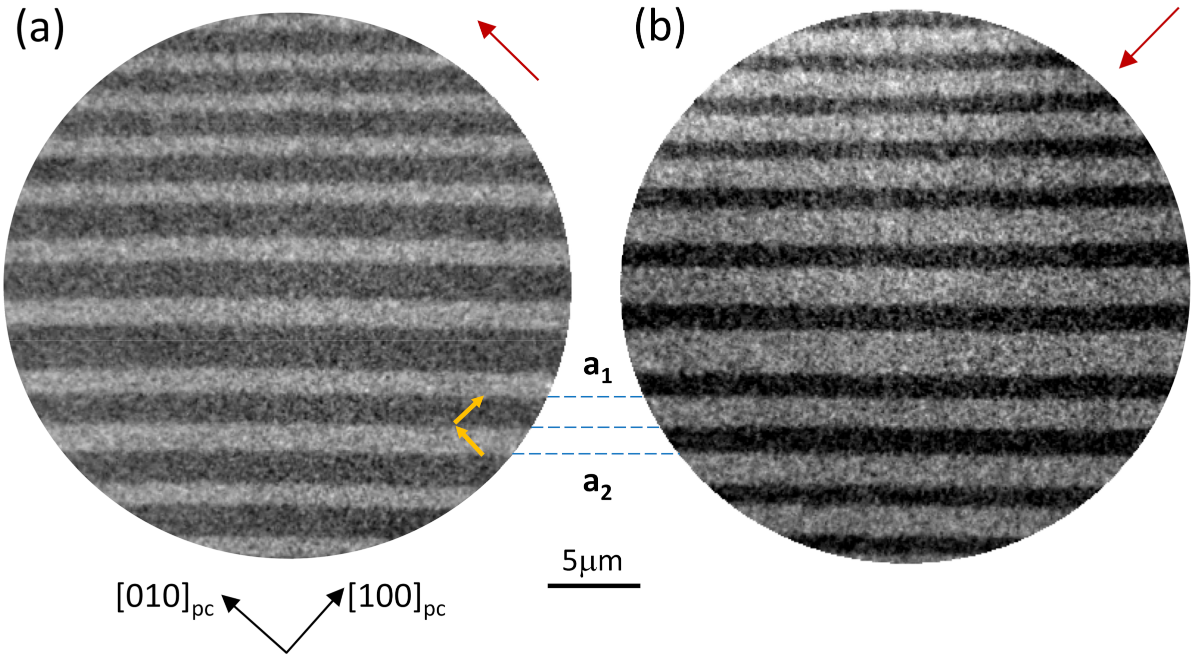

Figure SM 6. (a,b) XLD-PEEM images recorded at the Ti L_3-edge for two orthogonal incoming light directions. The in-plane projection of the incoming x-rays is indicated by the red arrows. The BTO pseudocubic crystallographic axes are shown in black with the yellow arrows indicating the local c-axis. Data from Sample A

V. VECTOR MAGNETIZATION MAPS

The XMCD (*xmcd*) is calculated, pixel by pixel, from the intensity (I) in a set of four images, recorded with left (l)/right (r) circular polarization on (res)/off (off) the Fe L_3 resonant photon energies as:

$$xmcd = \frac{I^r - I^l}{I^r + I^l}, \tag{5}$$

where

$$I^{r(l)} = \frac{I^{r(l)}_{res} - I^{r(l)}_{off}}{I^{r(l)}_{off}} = \frac{I^{r(l)}_{res}}{I^{r(l)}_{off}} - 1 \tag{6}$$

The XMCD is proportional to the projection of the magnetization, $\mathbf{M}$, along the x-ray photon propagation vector, $\mathbf{q}$, as

$$xmcd = \mathbf{M} \cdot \mathbf{q}, \tag{7}$$

The X-ray beam is incident on the sample surface at 16°. Under the assumption that the magnetisation lies in-plane, by acquiring two XMCD images with the sample rotated 90° about the sample normal (ϕ_s rotation), it is possible to probe locally the two components of $\mathbf{M}$ along the in-plane components $\mathbf{x}'$ and $\mathbf{y}'$ of the two orthogonal beam incoming directions:

$$\begin{cases} xmcd_{x'} = a \cdot M_{x'} \\ xmcd_{y'} = a \cdot M_{y'} \end{cases} \tag{8}$$

The ($\mathbf{x}'$, $\mathbf{y}'$) unit vectors define the frame of reference O', which is rotated by α_0 with respect to the image coordinate system O (see figure SM 7). The $(xmcd_{x'}, xmcd_{y'})$ are the measured XMCD on the two images and can be seen as the component of a *dichroism* vector $\mathbf{d} = xmcd_{x'}\mathbf{x}' + xmcd_{y'}\mathbf{y}'$, which is parallel to the in-plane magnetisation $\mathbf{M}$. The magnetisation angle α with respect to the image O polar coordinates and the dichroism magnitude can be retrieved as

$$\begin{cases} \alpha_M = atan\left(\frac{xmcd_{y'}}{xmcd_{x'}}\right) + \alpha_0 \\ |xmcd| = \sqrt{d^2_{x'} + d^2_{y'}} \end{cases} \tag{9}$$

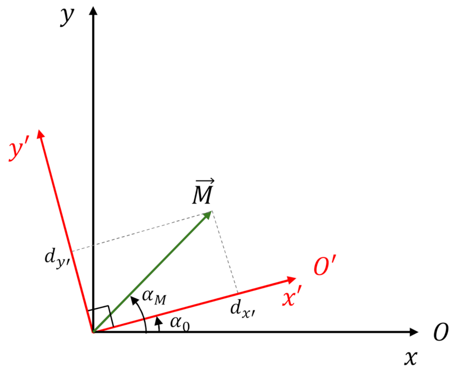

Figure SM 7. Schematic of the reference frame and angles used by the vector map formula. **M** is the in-plane magnetisation vector. The un-primed axes are the image coordinates, and the primed axes are parallel to the two mutually orthogonal in-plane x-ray propagation vectors of the two XMCD images.

Figure SM 8 (a) and (b) shows XMCD-PEEM images of the Fe FM domains for two orthogonal incoming light directions. The XMCD contrast is proportional to the projection of the local Fe magnetization onto the X-ray momentum (bright contrast indicates parallel alignment). The two XMCD images are distortion corrected, to achieve a best overlap, and then combined to form a vector map of the *in-plane* magnetisation (see Figure SM 8 (c)).

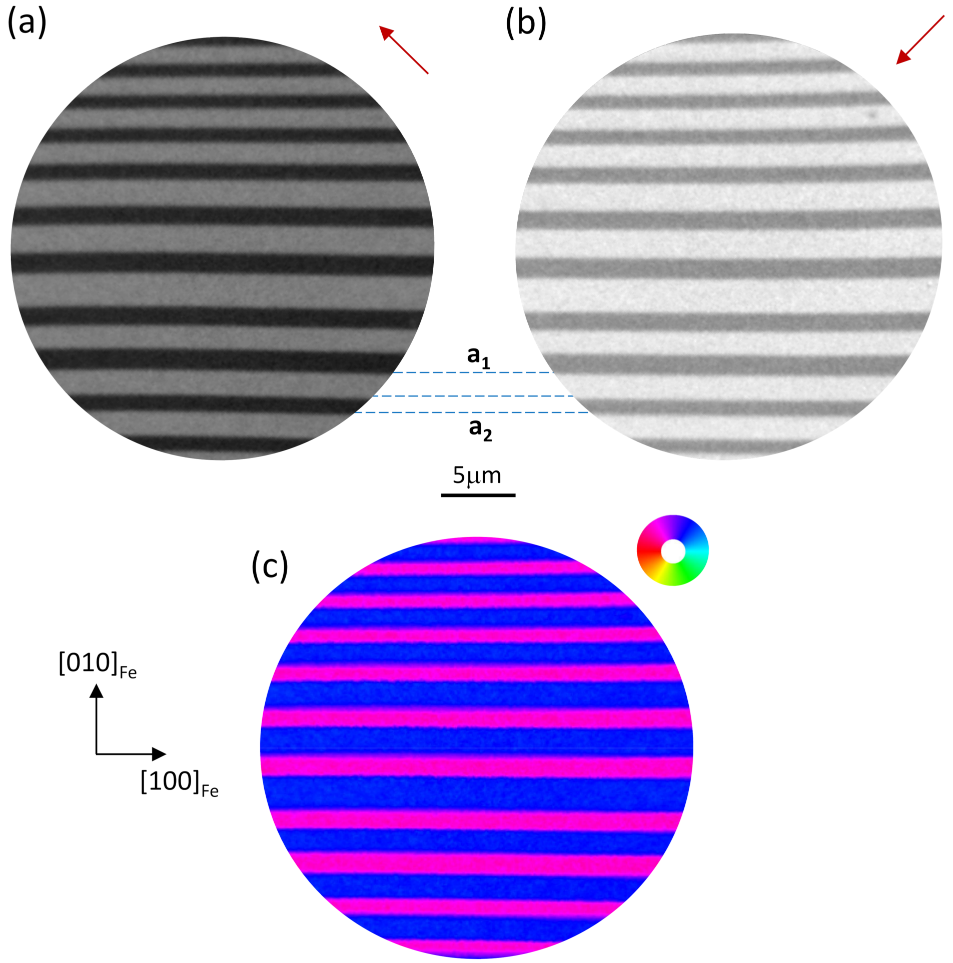

Figure SM 8. (a,b) XMCD-PEEM images recorded at the Fe L_3-edge for two orthogonal incoming light directions. The in-plane projection of the incoming x-rays is indicated by the red arrows. (c) Vector magnetisation map obtained from the combination of the two images. The magnetisation direction is represented by the colour wheel and the Fe crystallographic directions are shown in black. (Data from Sample A)

VI. MAGNETOELASTIC AND MAGNETOCRYSTALLINE ANISOTROPY IN bcc-Fe

The magnetic anisotropy free energy, F_{tot}, including magnetoelastic (ME) and magnetocrystalline (MC) terms implies a competition between uniaxial and biaxial contributions.

The magnetic free energy for the strained Fe film can be written as

$$F_{tot} = 2B_2\epsilon_{12}\alpha_1\alpha_2 + K_1\alpha_1^2\alpha_2^2 \tag{10}$$

where ϵ_{12} is a strain tensor component, B_2 is a first order MEL constant, K_1 is the first order MC constant and α_1 and α_2 are direction cosines of the in-plane (*i.e.* α_3=0) magnetisation, $\mathbf{M}$. The angle between $\mathbf{M}$ and $[100]_{Fe}$ is defined as γ. We can define a parameter

$$r = \frac{2B_2\epsilon_{12}}{K_1} \tag{11}$$

so that, for a a_1 domain (*i.e.* $\epsilon_{12} > 0$) and a bulk *bcc*-Fe MC anisotropy (*i.e.*$K_1 > 0$), stable minima are found for the conditions

$$\begin{cases} \dfrac{\partial F_{tot}}{\partial \gamma} = 0 \quad \Rightarrow \quad \cos(2\gamma)(1 + \dfrac{1}{r}\sin(2\gamma)) \ = \ 0 \\ \dfrac{\partial^2 F_{tot}}{\partial \gamma^2} > 0 \quad \Rightarrow \quad \cos(4\gamma) - r\sin(2\gamma) > \ 0 \end{cases} \tag{12}$$

with solutions

$$\begin{cases} r \leq -1 & \gamma = \dfrac{\pi}{4} + n\pi \\ -1 < r < 1 & \gamma = \dfrac{(-1)^n}{2}\arcsin(-r) + \dfrac{n\pi}{2} \\ r \geq 1 & \gamma = \dfrac{\pi}{4} + (2n+1)\pi \end{cases} \tag{13}$$

There are two regimes which are determined by the relative magnitude of B_2 and K_1. If $r > 1$ or $r < -1$, the magnetoelastic contribution dominates yielding a uniaxial anisotropy. Conversely, if $-1 < r < 1$, biaxial anisotropy develops as $r \to 0$. Figure SM 9 shows the evolution of F_{tot} from the biaxial case to the uniaxial case upon changing r.

For bulk *bcc*-Fe parameters, the magnetoelastic energy dominates (*i.e.* $r = 2.1$) so that the easy-axis aligns perpendicular to the local FEL *c*-axis [5]. If r is reduced from ± 1 to 0, two easy axes emerge along either the $[100]_{Fe}$ or $[010]_{Fe}$. Changing the magnitude B_2 therefore rotates the easy axis by 45°.

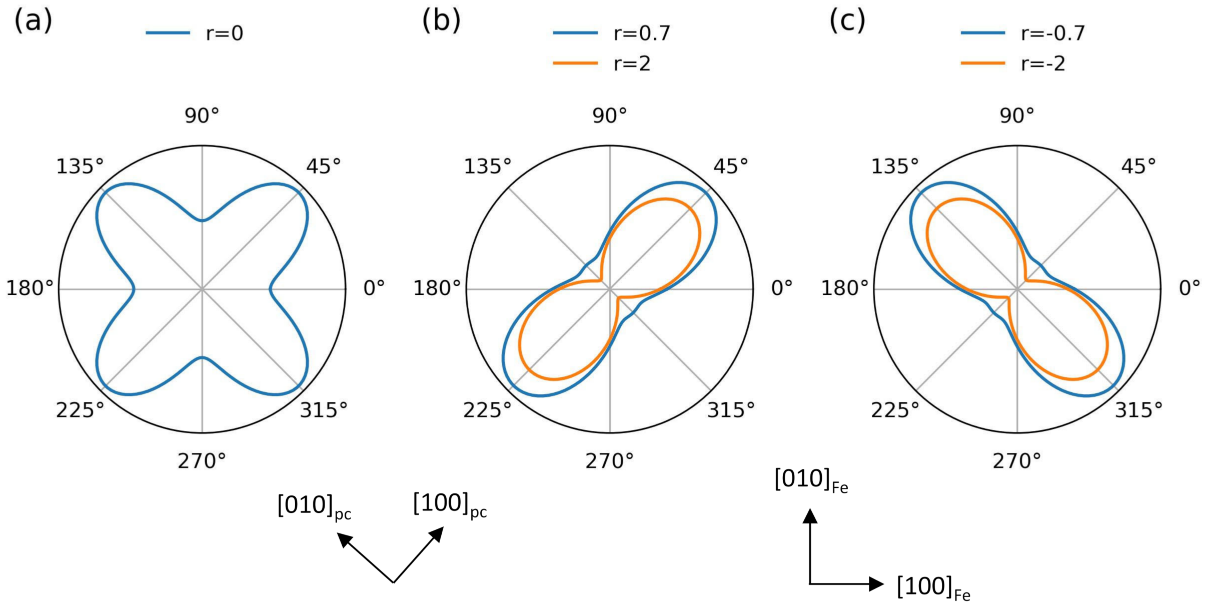

Figure SM 9. Polar plot of F_{tot} for different values of r. (a) Biaxial anisotropy with no shear-strain, (b) with shear strain uniaxial anisotropy for a a_1 FEL domain and (c) a_2 FEL domain.

VII. MICROMAGNETIC SIMULATIONS

The effects of M_{sat} on the FM domain configuration was explored by comparing simulated XMCD-PEEM images with the experimental XMCD-PEEM image (Figure SM 10). The simulations accurately reproduce the experimental XMCD-PEEM data for $1.5 < M_{sat} < 2.5\ MA/m$. Hence the bulk *bbc*-Fe value of $M_{sat} = 1.76\ MA/m$ was used for further simulations. The effects of B_2 and K_1 on the FM domain configuration were then explored by comparing micromagnetic simulation based XMCD-PEEM images with the experimental XMCD-PEEM results (see Figure SM 11). The simulations accurately reproduce the experimental XMCD-PEEM data for $B_2 = -3.93\ MJ/m^3 (K_1 = 0.042\ MJ/m^3)$ to $B_2 = -2\ MJ/m^3 (K_1 = 0.022\ MJ/m^3)$. Hence, $B_2 = -3 \pm 1\ MJ/m^3$.

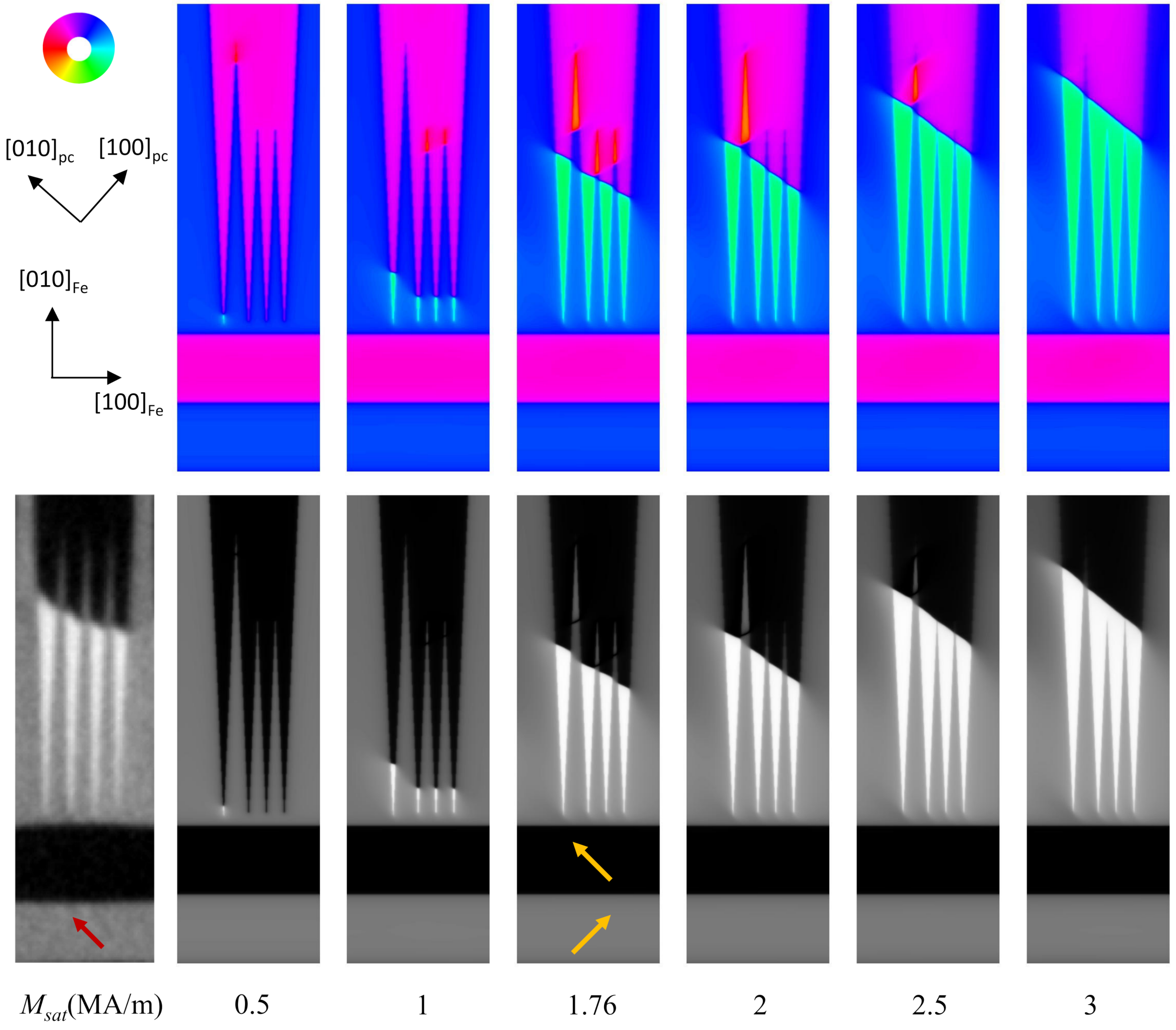

Figure SM 10. Micromagnetic simulations of the Fe domain structure as a function of M_{sat} (top row), the corresponding simulated XMCD-PEEM images (bottom row) and the experimental XMCD-PEEM image (bottom left). The FM domain wall across the the needle-like areas has a strong dependence on M_{sat}. The colour wheel indicates the magnetisation direction in the colour images. The red arrow indicates the in-plane projection of the incoming x-rays and the yellow arrows indicate the local BTO c-axis. The crystallographic directions are shown in black.

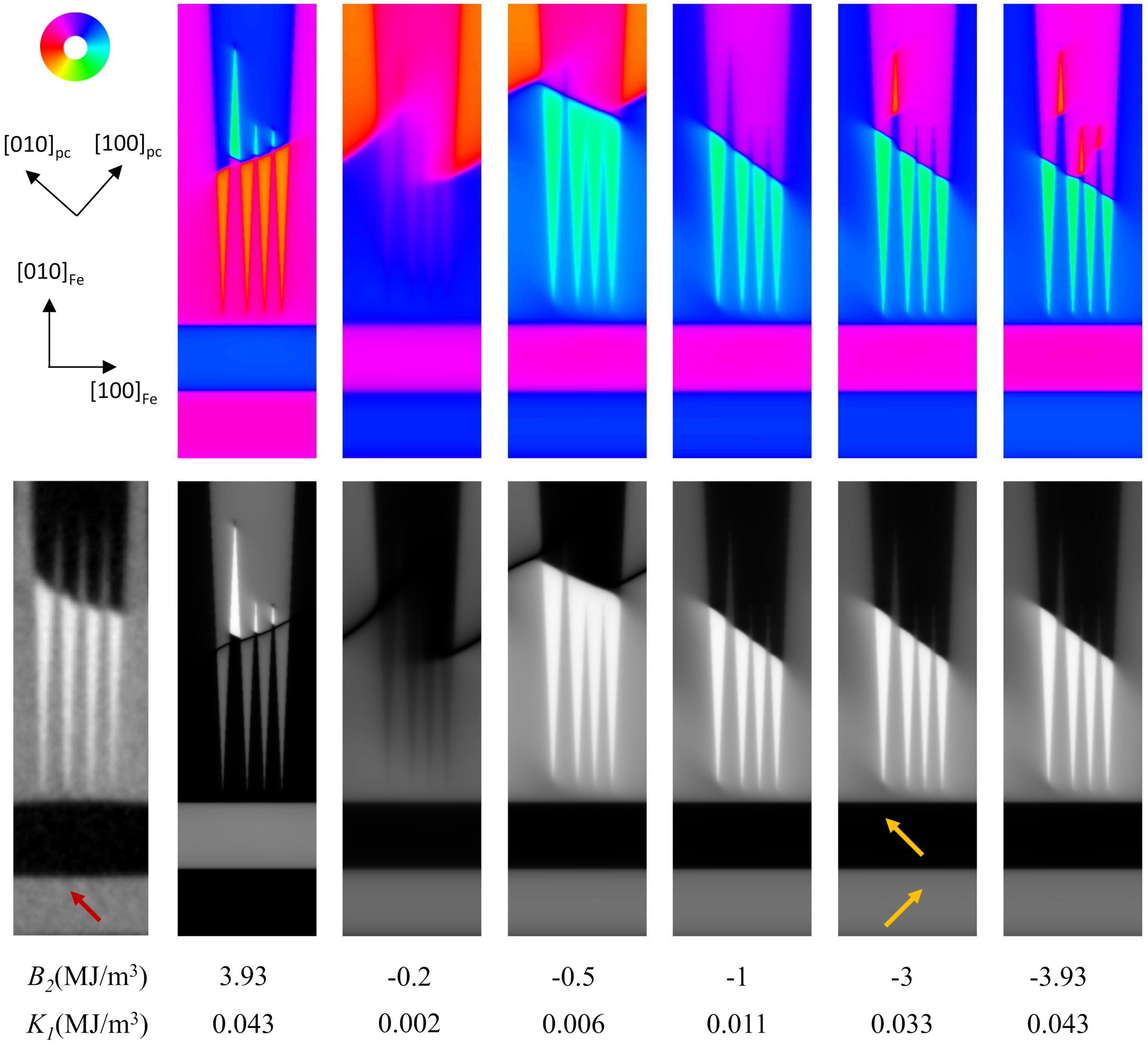

Figure SM 11. Micromagnetic simulations of the Fe domain structure as a function B_2 and K_1 for $r = -0.971$ (top row), corresponding simulated XMCD-PEEM images (bottom row) and the experimental XMCD-PEEM image (bottom left). The pattern transfer is partially lost if B_2 is below $-1\ MJ/m^3$. The colour wheel indicates the magnetisation direction in the colour images. The red arrow indicates the in-plane projection of the incoming x-rays and the yellow arrows indicate the local BTO c-axis. The crystallographic directions are shown in black

VIII. MAGNETOELASTIC FREE ENERGY WITH HIGHER ORDER TERMS

The stress in epitaxial *bcc*-Fe films can be described using higher order MEL coupling constants. It has been reported that the value of B_2 in epitaxial *bcc*-Fe films under stress can be described using higher order MEL coupling constants [6] so the the magnetoelastic free energy, F_{me}, can be expressed as

$$\begin{aligned} F_{me} &= B_1(\epsilon_{11}\alpha_1^2 + \epsilon_{22}\alpha_2^2 + \epsilon_{33}\alpha_3^2) \\ &+ 2B_2(\epsilon_{12}\alpha_1\alpha_2 + \epsilon_{23}\alpha_2\alpha_3 + \epsilon_{31}\alpha_3\alpha_1) \\ &+ \frac{1}{2}D_{11}(\alpha_1^4\epsilon_{11}^2 + \alpha_2^4\epsilon_{22}^2 + \alpha_3^4\epsilon_{33}^2) \\ &+ D_{12}(\alpha_1^2\alpha_2^2\epsilon_{11}\epsilon_{22} + \alpha_2^2\alpha_3^2\epsilon_{22}\epsilon_{33} + \alpha_3^2\alpha_1^2\epsilon_{33}\epsilon_{11}) \end{aligned} \tag{14}$$

where B_1, B_2, D_{11}, D_{12} are MEL coupling constants, ϵ is a stress tensor and the α terms are the directions cosines of the magnetisation axis.

Wedler *et al.* have determined the change in the magnetoelastic stress, σ, as the magnetization is rotated from the $[110]_{Fe}$ direction to the $[010]_{Fe}$ direction [6]. To interpret results from a cantilever technique [7], Wedler *et al.* included second order MEL terms to show that the change in the magnetoelastic stress can be explained by introducing an effective MEL coupling constant, $B_{2,eff}$, defined as

$$\sigma^{[110]} - \sigma^{[010]} = \frac{1}{2}B_2 + \frac{1}{4}(D_{12} - D_{11})\epsilon_0 \equiv \frac{1}{2}B_{2,eff} \tag{15}$$

where $\epsilon_0 = \epsilon_{11} = \epsilon_{22}$ is the average in-plane film stress.

The study found a sign reversal of $B_{2,eff}$ with increasing stress. On the other hand, the magnetic domain structure in the present study is not defined by the film stress but by the angular dependence of F_{me} which is negligibly affected by the second order corrections. F_{me} is a function of the magnetization direction and strain so that the sign reversal of $B_{2,eff}$ reported in [6] represents a different physical property to that related to the sign reversal of B_2 discovered in this study

By comparing the polar plot of the F_{me} up to first and second order (see Figure SM 12) we see that the easy-axis direction does not change. If the in-plane strain is increased to 11.2%, which is enough to reverse the sign of $B_{2,eff}$, F_{me} develops a biaxial anisotropy. The co-linear alignment of the Fe easy-axis with the BTO *c*-axis, cannot be explained by higher order corrections to F_{me} and arises from the sign reversal of B_2 revealed using PEEM.

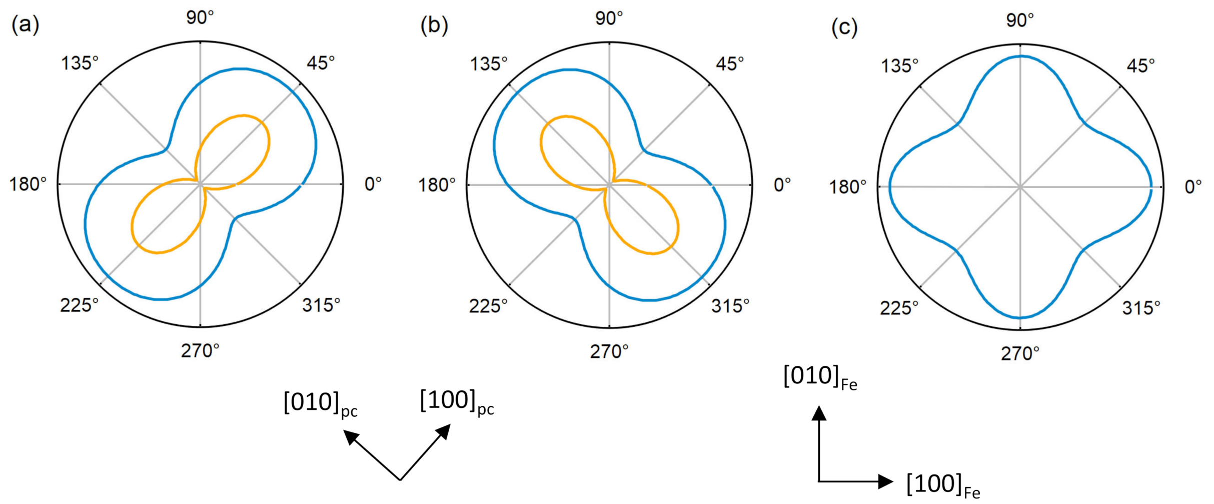

Figure SM 12. Polar plot of F_{me} up to first (orange) and second (blue) order for the (a) a_1 and (b) a_2 domains and (c) up to second order with a high *in-plane* stress ($\epsilon_{11} = \epsilon_{22} = 0.112$). The crystallographic directions are shown in black.

IX. CHANGES IN THE Fe FILM MAGNETISATION INDUCED BY POLING THE BTO SUBSTRATE

In order to further evaluate the dominant role of shear strain in determining the Fe film easy-axis, 1.5nm of Fe was deposited on a BaTiO substrate and capped with 2nm of Al to allow *in situ* poling. Two samples, referred to as sample B and sample C, were prepared. Figure SM13(a) shows an XLD-PEEM image of the FE domain structure and Figure SM13(b) shows an XMCD-PEEM image of the Fe FM domain structure recorded from sample B before poling. After poling (see Figure SM14(a) and (b)) a pattern of a_2 and c FEL domains appears which transfers as a single FM domain on the FEL a_1 and c domains. The angular distribution of $\mathbf{M}$ is shown in Figure SM13(c) and Figure SM14(c). The polar plot shown in Figure SM 9(c) predicts 4 possible domains in the presence of shear strain and Figure SM13(c) shows that three of these are observed in the XMCD-PEEM image from the unpoled sample B surface shown in Figure SM13(b). The angular position, γ, of the lobes is shown in Figure SM13(d) and Figure SM14(d) along with the stable minima (solid line) for the Fe easy-axis as a function of r. B_2 is then calculated using the different values of r determined for the plots shown in Figure SM13(d) and Figure SM14(d). Table SM 1 summarizes the results for the unpoled(poled) state of sample B and shows that the average value of B_2 for an a_2 FEL domain is $2.6 \pm 0.6(B_2 = 2.1 \pm 0.7)$ and remains unaffected by the poling. On the other hand, the c domain has an easy-axis close to the $[100]_{Fe}$ direction which is the expected Fe easy-axis in the absence of the shear strain uniaxial anisotropy. Similar results for Sample C are shown in Figure SM15 and Figure SM16 and summarised in Table SM 1.

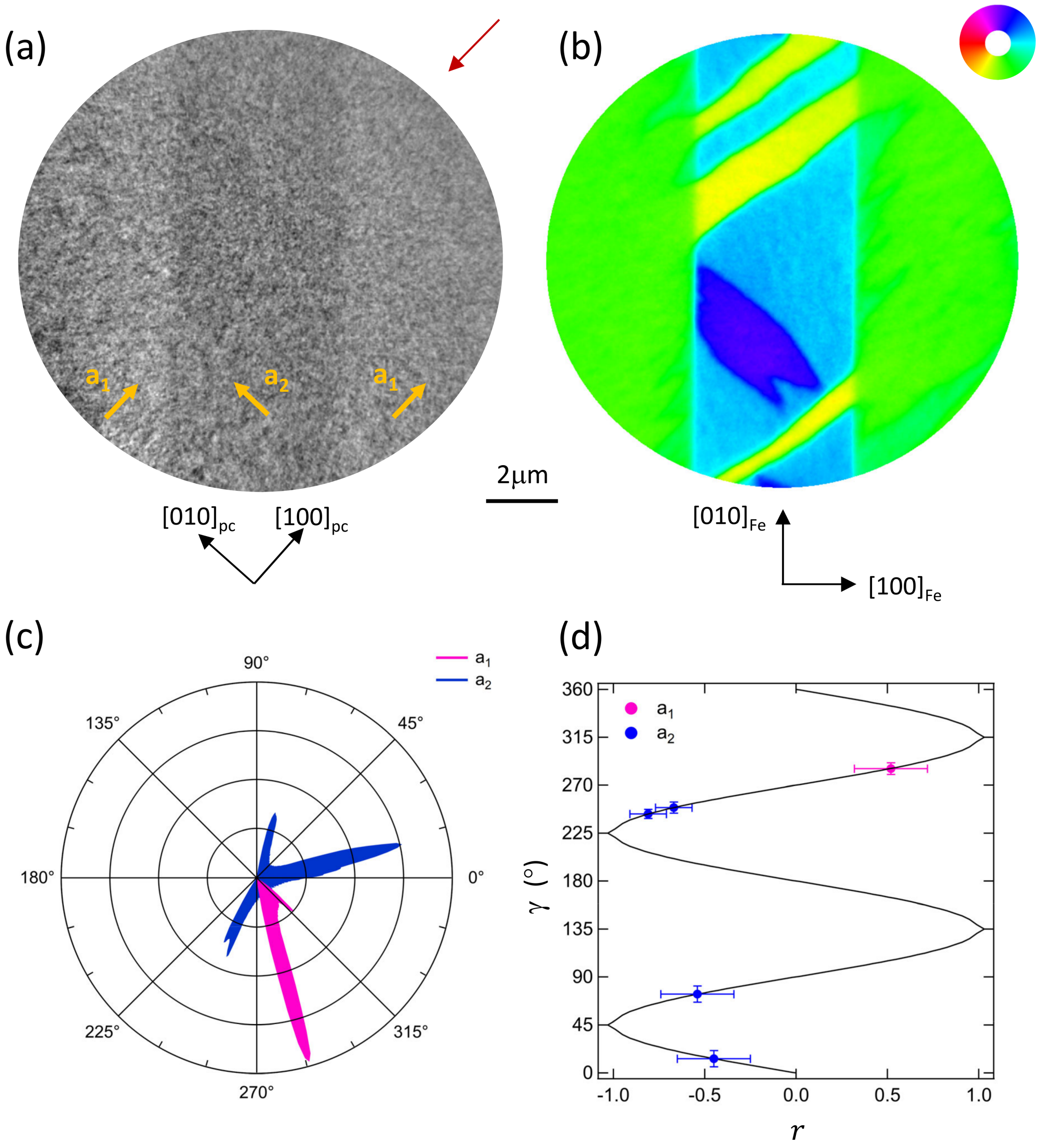

Figure SM 13. (a) XLD-PEEM of the FEL domains and (b) XMCD-PEEM vector map of the Fe magnetisation from an unpoled Al capped Fe/BTO sample. The red arrow represents the in-plane projection of the x-ray beam. The FEL domain c-axis orientation is indicated in yellow for each domain and the magnetisation direction is represented by the colour wheel. The crystallographic axes are shown in black. (c) Polar plot of the magnetisation angle (γ) distribution in (b). (d) γ as a function of r for stable minima of the magnetic free energy (solid black line). The experimental values of γ determined from the polar plot for the a_1/a_2 FEL domains are marked as the magenta/blue symbols. (Data from Sample B)

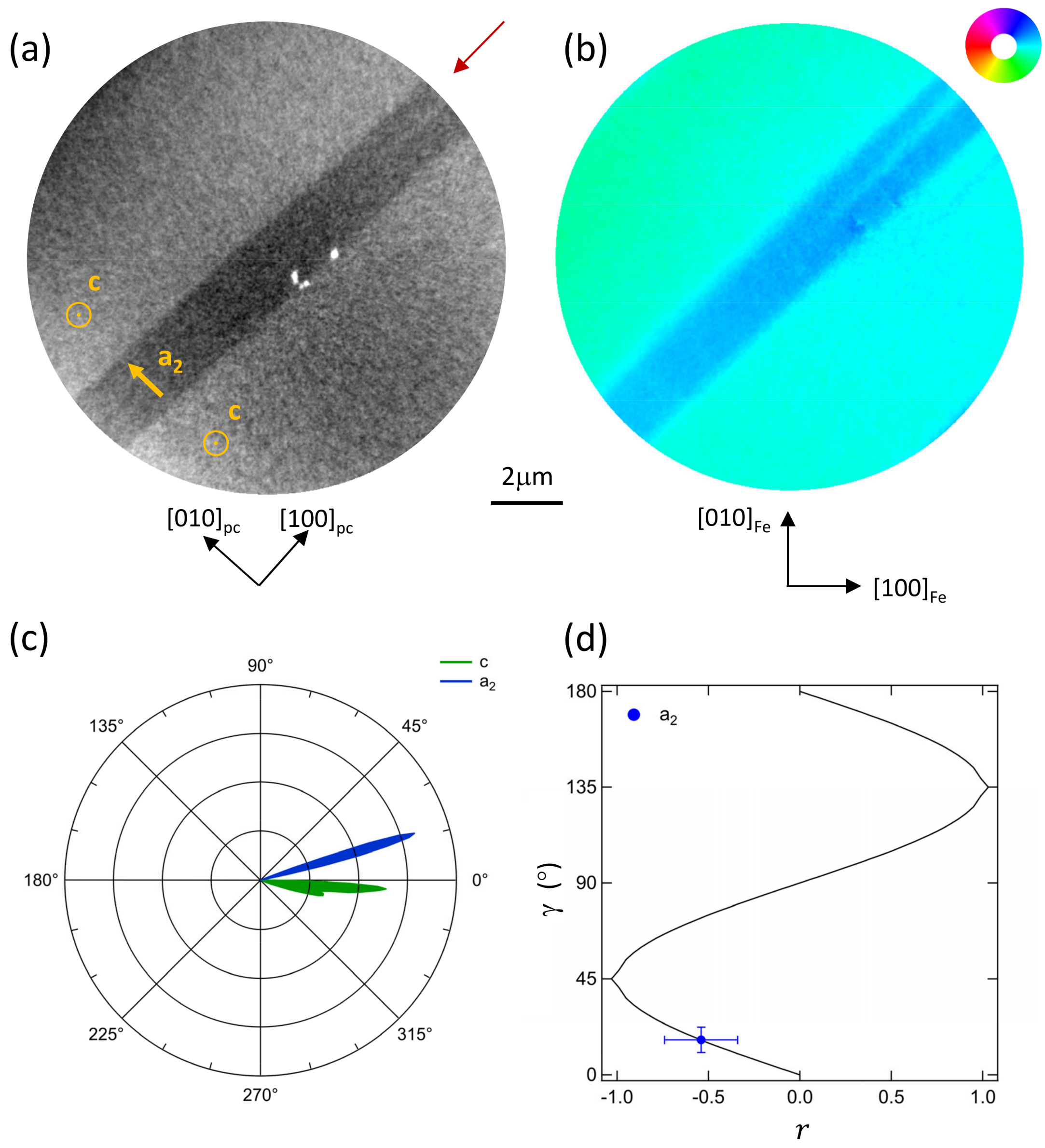

Figure SM 14. (a) XLD-PEEM of the FEL domains and (b) XMCD-PEEM vector map of the Fe magnetisation from the same sample as in Figure SM11, but after poling. The red arrow represents the in-plane projection of the x-ray beam. The FEL domain *c*-axis orientation is indicated in yellow for each domain and the magnetisation direction is represented by the colour wheel. The crystallographic axes are shown in black. (c) Polar plot of the magnetisation angle (γ) distribution in (b). (d) γ as a function of r for stable minima of the magnetic free energy (solid black line). The experimental values of γ determined from the polar plot for the a_1/a_2 FEL domains are marked as the magenta/blue symbols. (Data from Sample B)

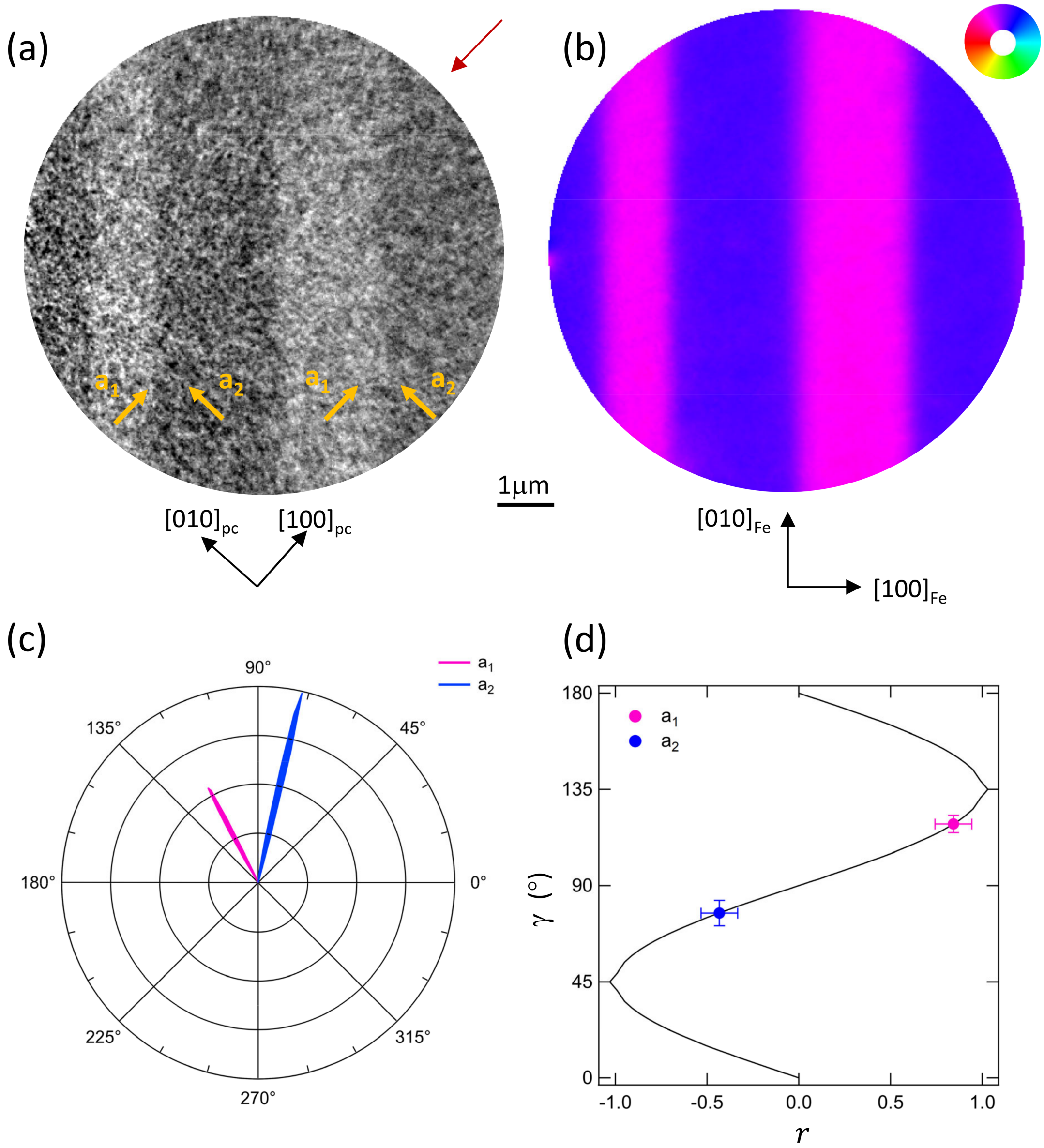

Figure SM 15. (a) XLD-PEEM of the FEL domains and (b) XMCD-PEEM vector map of the Fe magnetisation from an unpoled Al capped Fe/BTO sample. The red arrow represents the in-plane projection of the x-ray beam. The FEL domain c-axis orientation is indicated in yellow for each domain and the magnetisation direction is represented by the colour wheel. The crystallographic axes are shown in black. (c) Polar plot of the magnetisation angle (γ) distribution in (b). (d) γ as a function of r for stable minima of the magnetic free energy (solid black line). The experimental values of γ determined from the polar plot for the a_1/a_2 FEL domains are marked as the magenta/blue symbols. (Data from Sample C)

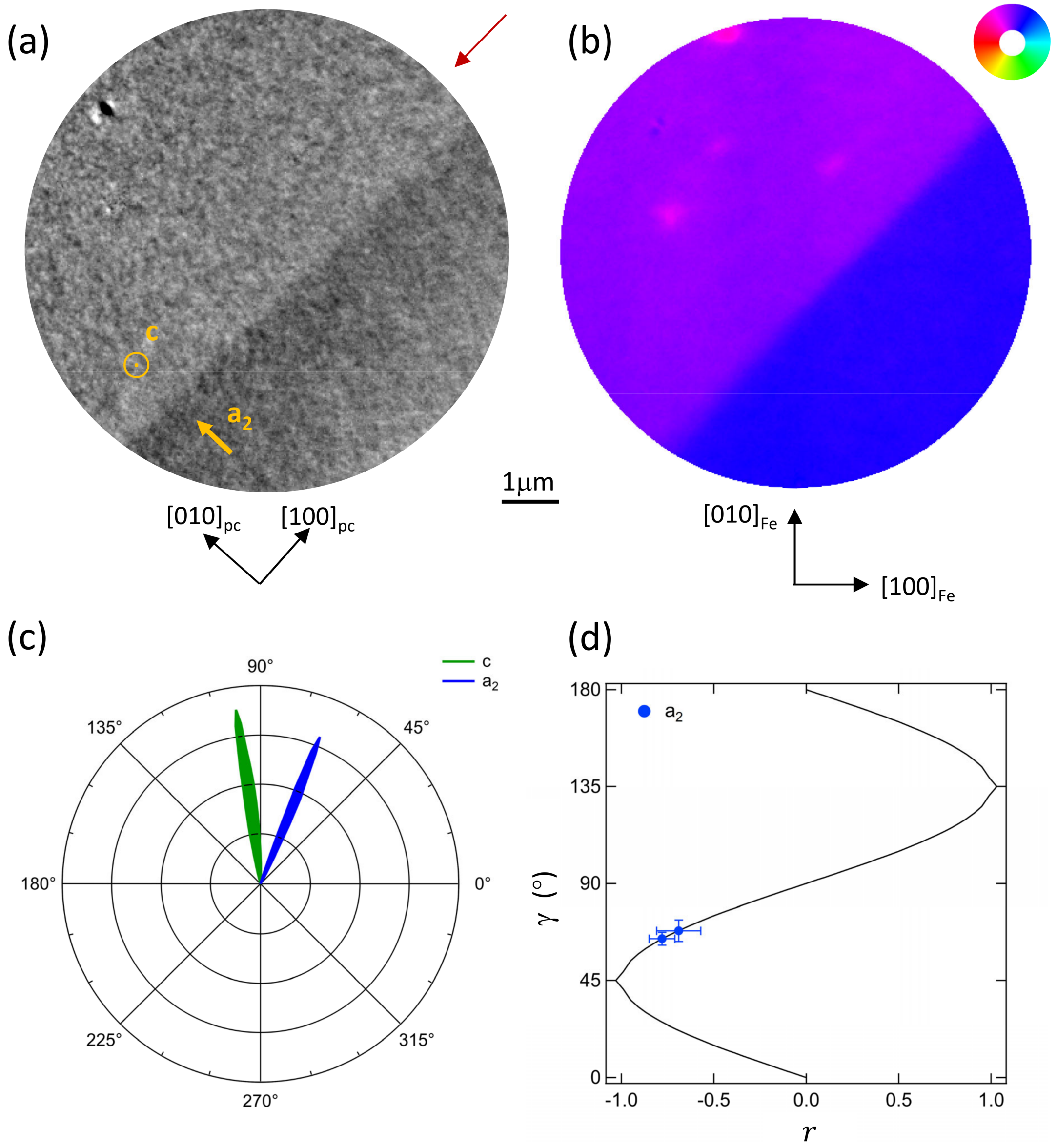

Figure SM 16. (a) XLD-PEEM of the FEL domains and (b) XMCD-PEEM vector map of the Fe magnetisation from the same sample as in Figure SM13, but after poling. The red arrow represents the in-plane projection of the x-ray beam. The FEL domain c-axis orientation is indicated in yellow for each domain and the magnetisation direction is represented by the colour wheel. The crystallographic axes are shown in black. (c) Polar plot of the magnetisation angle (γ) distribution in (b). (d) γ as a function of r for stable minima of the magnetic free energy (solid black line). The experimental values of γ determined from the polar plot for the a_1/a_2 FEL domains are marked as the magenta/blue symbols. (Data from Sample C)

**Sample B**				
**Unpoled**				
**domain**	$\mathbf{e_{1,2}}$	γ	**r**	$\mathbf{B_2}$**[MJ/m^3]**
a_2	-0.0055	243 ± 2	-0.8 ± 0.1	3.1 ± 0.3
a_2	-0.0055	249 ± 3	-0.7 ± 0.1	2.5 ± 0.5
a_2	-0.0055	13 ± 4	-0.5 ± 0.2	1.7 ± 0.9
a_2	-0.0055	74 ± 4	-0.5 ± 0.2	2.1 ± 0.8
a_1	+0.0055	286 ± 3	$+0.5 \pm 0.2$	2.0 ± 0.6
				$\overline{B}_2 = \mathbf{2.6 \pm 0.6}$
**Poled**				
**domain**	$\mathbf{e_{1,2}}$	γ	**r**	$\mathbf{B_2}$**[MJ/m^3]**
c	0	355 ± 4	-	-
a_2	-0.0055	16 ± 3	-0.5 ± 0.2	2.1 ± 0.7
				$B_2 = \mathbf{2.1 \pm 0.7}$

**Sample C**				
**Unpoled**				
**domain**	$\mathbf{e_{1,2}}$	γ	**r**	$\mathbf{B_2}$**[MJ/m^3]**
a_2	-0.0055	77 ± 1	-0.4 ± 0.1	1.7 ± 0.4
a_1	+0.0055	119 ± 2	$+0.8 \pm 0.1$	3.2 ± 0.5
				$\overline{B}_2 = \mathbf{2.4 \pm 0.8}$
**Poled**				
**domain**	$\mathbf{e_{1,2}}$	γ	**r**	$\mathbf{B_2}$**[MJ/m^3]**
c	0	96 ± 3	-	-
c	0	98 ± 3	-	-
a_2	-0.0055	64 ± 1	-0.8 ± 0.1	3.0 ± 0.3
a_2	-0.0055	68 ± 2	-0.7 ± 0.1	2.6 ± 0.5
				$\overline{B}_2 = \mathbf{2.8 \pm 0.2}$

Table SM I. Local magnetisation angle,γ, and r extracted from the polar plots, shown in Figures SM11-14(c), for samples B and C together with the corresponding values of B_2 and the average value of B_2 for the unpoled and poled states. The error in γ represents the FWHM of the polar plot peak which propagates to r and B_2.

X. FERROMAGNETIC RESONANCE

Figure SM 17 shows the in-plane angular dependent (φ_H) FMR spectrum from Sample D, Fe(1.5nm) / BTO(001) capped with 2nm of Aluminium, acquired at f = 16 GHz. Two modes are observed that are rotated by $\sim 90\,^\circ$ from each other, corresponding to the a_1 and a_2 domains.

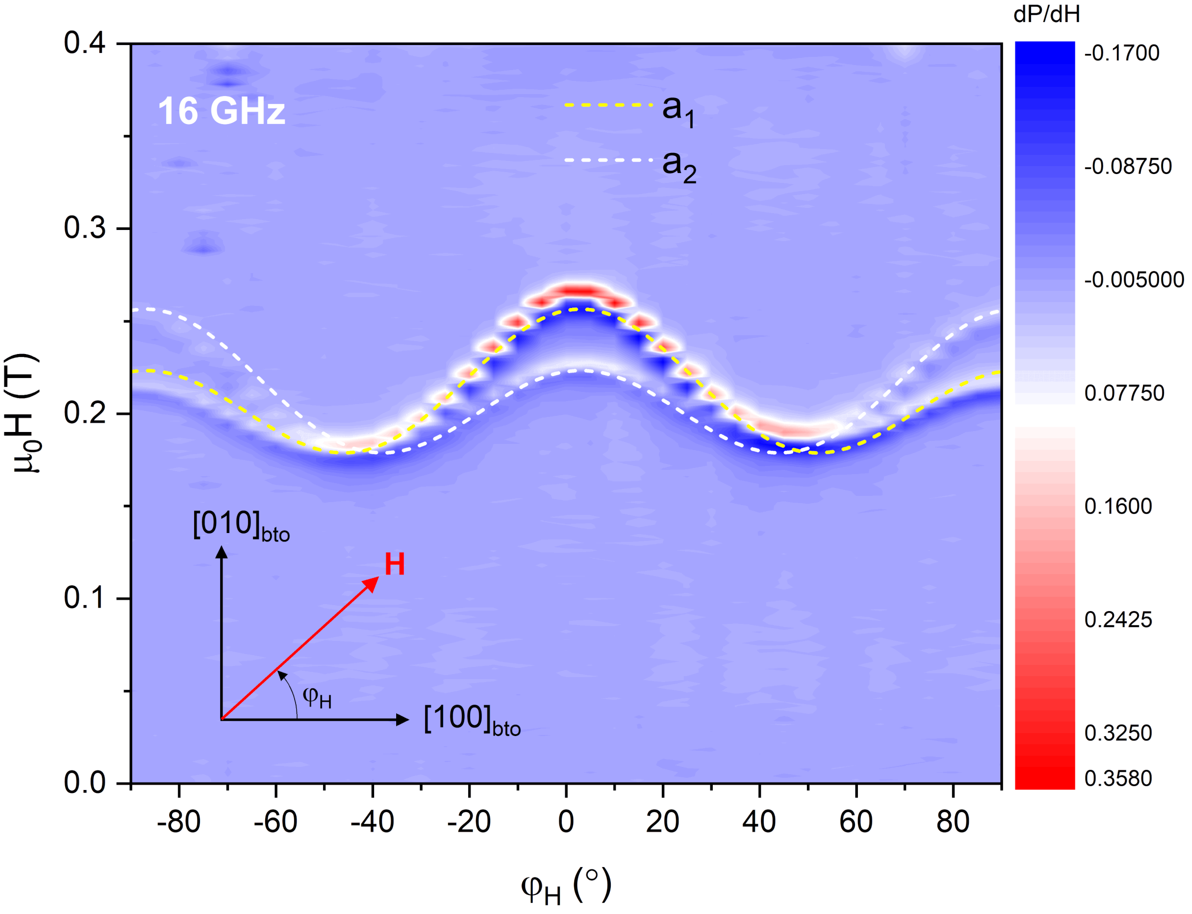

Figure SM 17. FMR spectrum from sample D (color plot) with the simulated FMR dispersions for a_1/a_2 domains shown as yellow/white dashed lines.

An expression for the FMR angular frequency is obtained from the Smith and Beljers equation as shown in [8]

$$\left(\frac{\omega}{\gamma}\right)^2 = \frac{1}{M^2 sin^2\theta}\left[\frac{\partial^2 F}{\partial\theta^2}\frac{\partial^2 F}{\partial\varphi^2} - \left(\frac{\partial^2 F}{\partial\theta\partial\varphi}\right)^2\right] \tag{16}$$

where M is the saturation magnetization, ω the microwave angular frequency, γ the gyromagnetic ratio and θ and φ are the polar and in-plane azimuthal angle of the magnetization, respectively. The magnetic free energy density, F, is an expression similar to Eq.(5) but with additional terms to account for the Zeeman and demagnetizing fields. For the experimental conditions used we take $\theta = \pi/2$ and make the approximation that $\varphi = \varphi_H$ i.e. the magnetization is aligned in-plane with the applied magnetic field (H); a valid approximation given $H > H_{K_1}, H_\epsilon$. After obtaining an expression for $\omega(H, \varphi, \epsilon)$ and applying the above conditions, we then solve the quadratic to obtain $H_r(\omega, \varphi_H, \epsilon)$, where H_r is the resonant field. Fits of $H_r(\omega, \varphi_H, \epsilon)$ to the experimental data for a_1 and a_2 domains, shown by the yellow and white dashed lines in Figure SM 17, give $M \approx 1.7$ MAm^{-1}, $K_1 \approx 30$ kJm^{-3}, $\epsilon = \pm\ 0.005$ and $B_2 \approx 1.5$ MJm^{-3}, similar to the values obtained from the XMCD-PEEM data.

[1] S. Cherifi, R. Hertel, S. Fusil, H. Béa, K. Bouzehouane, J. Allibe, M. Bibes, and A. Barthélémy, physica status solidi (RRL) – Rapid Research Letters **4**, 22 (2010), https://onlinelibrary.wiley.com/doi/pdf/10.1002/pssr.200903297.

[2] R. V. Chopdekar, V. K. Malik, A. Fraile Rodríguez, L. Le Guyader, Y. Takamura, A. Scholl, D. Stender, C. W. Schneider, C. Bernhard, F. Nolting, and L. J. Heyderman, Phys. Rev. B **86**, 014408 (2012).

[3] J. E. Rault, T. O. Menteş, A. Locatelli, and N. Barrett, Scientific Reports **4**, 6792 (2014).

[4] M. W. Haverkort, M. Zwierzycki, and O. K. Andersen, Phys. Rev. B **85**, 165113 (2012).

[5] T. H. E. Lahtinen, Y. Shirahata, L. Yao, K. J. A. Franke, G. Venkataiah, T. Taniyama, and S. van Dijken, Applied Physics Letters **101**, 262405 (2012), https://doi.org/10.1063/1.4773482.

[6] G. Wedler, J. Walz, A. Greuer, and R. Koch, Surface Science **454-456**, 896 (2000).

[7] M. Weber, R. Koch, and K. H. Rieder, Phys. Rev. Lett. **73**, 1166 (1994).

[8] M. Farle, Reports on Progress in Physics **61**, 755 (1998).
